# Prevalence of and Risk Factors for Extremely Low Birth Weight Infants in Saudi Arabia: A Four-Year Single-Center Experience

**DOI:** 10.7759/cureus.85202

**Published:** 2025-06-01

**Authors:** Mohammad Alhasoon

**Affiliations:** 1 Department of Pediatrics, College of Medicine, Qassim University, Buraydah, SAU

**Keywords:** extremely low birth weight, neonatal intensive care unit, preterm infant, prevalence, risk factors, triplets, twins

## Abstract

Introduction

Globally, extremely low birth weight (ELBW) infants present a distinct challenge for neonatologists, representing a significant portion of the most vulnerable and high-risk cases admitted to the neonatal intensive care units (NICUs). Saudi Arabia has a high prevalence of ELBW, which leads to a significant risk of morbidity and mortality, as many of these extreme preterm infants who are admitted to NICUs may not survive till discharge. This study aimed to evaluate the prevalence of ELBW infants and associated risk factors over a four-year period.

Methods

Two study designs were used in this research: a cross-sectional retrospective design was used for estimating the prevalence rate over four years, and a case-control study with a retrospective chart review for the risk factor correlation. The study was conducted at King Abdulaziz Medical City (KAMC), Riyadh, Kingdom of Saudi Arabia, covering four years, from January 2017 to December 2020. All newborn live infants born at KAMC who weighed 1000 grams or less were included in the study. Infants born outside KAMC and those with fatal anomalies were excluded from the study. The pregnancy notes on the mothers were reviewed blindly, with no knowledge of the newborn's outcome. Statistical data were analyzed using SPSS version 26.

Results

A total of 256 ELBW infants were recorded out of 36,000 live births. The prevalence of ELBW during the study period of four years was 0.71%. Of the ELBW infants, 57.4% had a birth weight between 750 and 1000 grams, while 42.6% weighed less than 750 grams. Lower gestational age and prolonged rupture of membrane (PROM) were associated with a birth weight of 750-1000 g, whereas twin and triplet births were associated with a birth weight of <750 g. Interestingly, there was a positive correlation between birth weight and maternal age, but birth weight was inversely correlated with gestational age.

Conclusion

There was a minimal incidence rate of ELBW during the four-year duration. Significant risk factors for birth weight <750 g were triplets and twins, while lower gestational age and PROM were significant risk factors for birth weight of 750-1000 g. Interventions focusing on improving antenatal care access, maternal health education, and nutritional status may help reduce the prevalence of ELBW in our region.

## Introduction

Around the world, extremely low birth weight (ELBW) accounts for a total of 1.2-1.5% of all live births and 15-20% of admissions to neonatal intensive care units (NICUs) [1]. Prematurity accounts for approximately 11.1% of all pregnancies and births; however, the survival rates of ELBW infants have significantly increased over the past half-century [2,3]. Improvements in neonatal intensive care helped to lower the rates of morbidity and death. 

Nonetheless, the high rate of ELBW births in Saudi Arabia poses significant mortality risks, as nearly one-third of preterm infants admitted to NICUs do not survive to discharge [4]. There is a dearth of trustworthy quantitative data highlighting the prevalence of ELBW newborns in Saudi Arabia and the risk factors that are linked to them. A study analysed the differences in survival rates between 1994 and 2019 [1]; however, few recent studies have investigated which specific intervention strategies for preterm newborns are most effective in reducing prevalence rates or mitigating the associated risks [5-7]. Performance standards could be set that obstetricians can use to track changes in local institutions' rates of morbidity and death. However, while analyzing the immediate and long-term health consequences of premature births [8], researchers must also examine the number of preterm births resulting in ELBW. Given that ELBW frequently leads to major health issues after birth, its epidemiological ramifications are so great that there is a dire need for additional empirical data to assess the maternal and perinatal factors influencing ELBW. Also, the paucity of literature indicates that there must be more evidence-based suggestions for better clinical judgment. More focus on regional and local differences in ELBW prevalence rates is necessary to close these gaps. To record how Saudi Arabian women manage the risks of preterm and mortality, survey or interview data must be gathered.

## Materials and methods

The study was conducted at King Abdulaziz Medical City (KAMC), Riyadh, Kingdom of Saudi Arabia, covering four years, from January 2017 to December 2020. KAMC is a single hospital with one NICU that includes three levels of care: Level III with 40 beds, Level II (also referred to as the Intermediate Care Nursery) with 36 beds, and Level I, which is the regular nursery. The hospital has an average of approximately 9,000 deliveries per year, including both newborns needing care and admitted to the NICU and healthy newborns who room-in with their mothers. Two study designs were used in this study: (i) a cross-sectional retrospective design was used to estimate the prevalence rate over the four years, and (ii) a case-control design with retrospective chart review for correlating the associated risk factors. 

All live-born infants weighing 1000 g or less at birth, delivered at KAMC, were included in the study. Infants born outside KAMC and those with fatal anomalies like Trisomies 13, 18, and major congenital anomalies like anencephaly were excluded from the study. The pregnancy notes on the mothers were reviewed blindly without knowledge of the newborn outcome. 

More than 10 variables were assessed, including demographic data and perinatal events such as maternal age, parity, maternal diabetes, maternal hypertension/preeclampsia, prolonged premature rupture of membranes (PPROM) defined as “rupture of membranes more than 18 hours before delivery”, the use of antenatal steroids, spontaneous pregnancy or assisted conception, number of fetuses, birth weight, gestational age, and sex of newborn.

Statistical analysis

The data were analyzed using IBM SPSS Statistics for Windows, version 26 (Released 2019; IBM Corp., Armonk, New York, United States). Descriptive statistics were given as numbers and percentages (%) for all categorical variables. The association of sex and birth weight with other measured variables was analyzed using the Chi-square test. In addition, the Pearson correlation coefficient was performed to determine the correlation between maternal age and gestational age according to birth weight. A p-value of less than 0.05 was considered significant.

Ethical concerns

The King Abdullah International Medical Research Centre (KAIMRC) institutional review board approved the study (approval number: RC20/283/R) and waived the patient consent as the study design was a retrospective chart review and no identifiable patient data were used. The study was carried out in accordance with relevant guidelines and regulations and the Declaration of Helsinki.

## Results

Out of a total of 36,000 newborns over the study period of four years, 256 were classified as ELBW. The prevalence of ELBW during this period was 0.71%. As seen in Table 1, 22.7% of the pregnancies had a gestational age between 23 and 26 weeks. More than half (52%) had a maternal age of 30 years or less. Approximately 60.2% had 1-2 parities. The most preferred mode of delivery was cesarean section (80.1%). A total of 187 (73.0%) underwent the complete course of antenatal steroids. The most common maternal factor was PPROM (17.6%). Chorionicity was mostly dizygotic dichorionic diamniotic (44.5%). Spontaneous conception was recorded in 54.7%. Triplet fetuses constituted 57.8% of the ELBW births, and mortality rates accounted for 12.9%. When comparing male and female newborns, it was observed that the difference between sexes did not reach statistical significance for any of the measured characteristics (p>0.05 for all variables).

**Table 1:** Association of the study variables with the sex of ELBW newborns (N=256) § P-value was calculated using Chi-square test; *outcome of death was defined as the baby passing away before hospital discharge DC: dichorionic; DA: diamniotic; MC: monochorionic; MA: monoamniotic; ELBW: extremely low birth weight; PPROM: prolonged premature rupture of membranes; NSVD: normal spontaneous vaginal delivery

Study variables	Total, n (%)	Gender		P-value ^§^
		Male (n=145), n (%)	Female (n=111), n (%)	
Gestational age				
23 - 26 weeks	58 (22.7%)	32 (22.1%)	26 (23.4%)	0.469
27 - 29 weeks	43 (16.8%)	28 (19.3%)	15 (13.5%)	
30 - 33 weeks	155 (60.5%)	85 (58.6%)	70 (63.1%)	
Maternal age				
≤30 years	133 (52.0%)	77 (53.1%)	56 (50.5%)	0.674
>30 years	123 (48.0%)	68 (46.9%)	55 (49.5%)	
Parity				
None	56 (21.9%)	30 (20.7%)	26 (23.4%)	0.772
1 – 2	154 (60.2%)	90 (62.1%)	64 (57.7%)	
>2	46 (18.0%)	25 (17.2%)	21 (18.9%)	
Mode of delivery				
NSVD	51 (19.9%)	23 (15.9%)	28 (25.2%)	0.063
Cesarean section	205 (80.1%)	122 (84.1%)	83 (74.8%)	
Antenatal steroid use				
No	32 (12.5%)	21 (14.5%)	11 (09.9%)	0.539
Incomplete course	37 (14.5%)	21 (14.5%)	16 (14.4%)	
Complete course	187 (73.0%)	103 (71.0%)	84 (75.7%)	
Maternal comorbities				
Maternal hypertension/preeclampsia	27 (10.5%)	15 (10.3%)	12 (10.8%)	0.904
Maternal diabetes	40 (15.6%)	23 (15.9%)	17 (15.3%)	0.905
PPROM (>18 hours)	45 (17.6%)	28 (19.3%)	17 (15.3%)	0.704
Chorionicity				
Singleton	27 (10.5%)	14 (09.7%)	13 (11.7%)	0.758
Dizygotic DC DA	114 (44.5%)	66 (45.5%)	48 (43.2%)	
Monozygotic DC DA	38 (14.8%)	23 (15.9%)	15 (13.5%)	
Monozygotic MC DA	03 (01.2%)	01 (0.70%)	02 (01.8%)	
Monozygotic MC MA	56 (21.9%)	30 (20.7%)	26 (23.4%)	
Triplets	12 (04.7%)	06 (04.1%)	06 (05.4%)	
Spontaneous or assisted conception				
Spontaneous	140 (54.7%)	74 (51.0%)	66 (59.5%)	0.180
Assisted	116 (45.3%)	71 (49.0%)	45 (40.5%)	
Number of fetuses				
Twins	34 (13.3%)	20 (13.8%)	14 (12.6%)	0.692
Triplets	148 (57.8%)	84 (57.9%)	64 (57.7%)	
Quadruplets	56 (21.9%)	30 (20.7%)	26 (23.4%)	
Quintuplets	12 (04.7%)	06 (04.1%)	06 (05.4%)	
Sextuplets	06 (02.3%)	05 (03.4%)	01 (0.90%)	
Outcome				
Death*	33 (12.9%)	16 (11.0%)	17 (15.3%)	0.311
Alive	223 (87.1%)	129 (89.0%)	94 (84.7%)	

Table 2 presents a comparison of various maternal, fetal, and perinatal characteristics between two groups of neonates with extremely low birth weights: those weighing 750-1000 g and those weighing less than 750 g. Several statistically significant associations were observed. It shows that increasing gestational age (p<0.001) and PPROM (p<0.001) were more strongly associated with birth weight of 750-1000 g, whereas birth weight under 750 g was more closely linked to dizygotic DC DA (p<0.001) and triplet pregnancies (p<0.001). Other variables such as sex, maternal age, parity, mode of delivery, maternal comorbidities (hypertension, diabetes), use of antenatal steroids, and method of conception (spontaneous vs. assisted) were not significantly associated with differences in birth weight between the two groups. Though a higher proportion of male infants was observed in the <750 g group (63.3% vs. 51.7% in the 750-1000 g group), this did not reach statistical significance (p=0.064). Finally, while a higher mortality rate was observed in the 750-1000 g group (16.3%) compared to the <750 g group (8.3%), this difference was not statistically significant (p=0.057), possibly due to improved survival rates with intensive neonatal care.

**Table 2:** Association of study variables with birth weight (N=256) § P-value was calculated using Chi-square test; *outcome of death was defined as the baby passing away before hospital discharge; ** Significant at p<0.05 level DC: dichorionic; DA: diamniotic; MC: monochorionic; MA: monoamniotic; ELBW: extremely low birth weight; PPROM: prolonged premature rupture of membranes; NSVD: normal spontaneous vaginal delivery

Study Variables	Birth weight		P-value ^§^
	750-1000 g (n=147), n (%)	<750 g (n=109), n (%)	
Sex			
Male	76 (51.7%)	69 (63.3%)	0.064
Female	71 (48.3%)	40 (36.7%)	
Gestational age			
23 - 26 weeks	45 (30.6%)	13 (11.9%)	<0.001 **
27 - 29 weeks	12 (08.2%)	31 (28.4%)	
30 - 33 weeks	90 (61.2%)	65 (59.6%)	
Maternal age			
≤30 years	69 (46.9%)	64 (58.7%)	0.062
>30 years	78 (53.1%)	45 (41.3%)	
Parity			
None	27 (18.4%)	29 (26.6%)	0.148
1 – 2	89 (60.5%)	65 (59.6%)	
>2	31 (21.1%)	15 (13.8%)	
Mode of delivery			
NSVD	29 (19.7%)	22 (20.2%)	0.928
Cesarean section	118 (80.3%)	87 (79.8%)	
Maternal comorbidities			
Maternal hypertension/preeclampsia	15 (10.2%)	12 (11.0%)	0.836
Maternal diabetes	26 (17.7%)	14 (12.8%)	0.291
PPROM (>18 hours)	32 (21.8%)	13 (11.9%)	<0.001 **
Antenatal steroids use			
No	20 (13.6%)	12 (11.0%)	0.825
Incomplete course	21 (14.3%)	16 (14.7%)	
Complete course	106 (72.1%)	81 (74.3%)	
Chorionicity			
Singleton	27 (18.4%)	0	<0.001 **
Dizygotic DC DA	52 (35.4%)	62 (56.9%)	
Monozygotic DC DA	16 (10.9%)	22 (20.2%)	
Monozygotic MC DA	02 (01.4%)	01 (0.90%)	
Monozygotic MC MA	38 (25.9%)	18 (16.5%)	
Triplets	12 (08.2%)	0	
Quintuplets	0	06 (05.5%)	
Spontaneous pregnancy or assisted conception			
Spontaneous	80 (54.4%)	60 (55.0%)	0.921
Assisted	67 (45.6%)	49 (45.0%)	
Number of fetuses			
Twins	34 (23.1%)	0	<0.001 **
Triplets	63 (42.9%)	85 (78.0%)	
Quadruplets	38 (25.9%)	18 (16.5%)	
Quintuplets	12 (08.2%)	0	
Sextuplets	0	06 (05.5%)	
Outcome			
Death	24 (16.3%)	09 (08.3%)	0.057
Alive	123 (83.7%)	100 (91.7%)	

**Figure 1:**
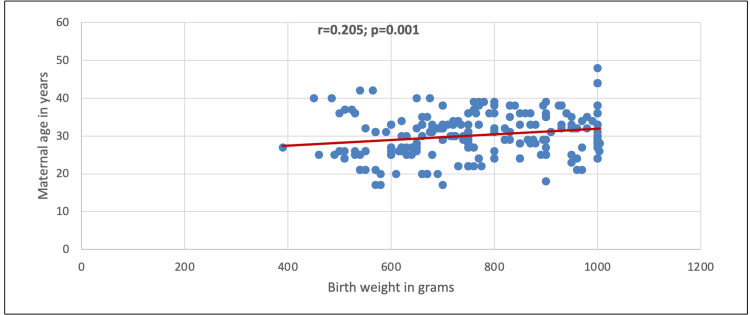
Correlation between birth weight and maternal age

In Figure 1, a statistically significant positive correlation between birth weight and maternal age was observed (r=0.205; p=0.001).

## Discussion

This study investigated the prevalence of ELBW and the risk factors for it in a tertiary healthcare setting. The prevalence of ELBW in the four-year study period was found to be 0.71% (256 out of 36000 baby births). This is almost consistent with a study conducted in Bahrain, which reported an incidence of very low birth weight (VLBW) of 0.52% over a 10-year period (August 1986 to July 1996) [9]. Similarly, a study done in Jeddah found that the prevalence of very low birth weight (VLBW) in three maternity and children hospitals in Jeddah City was 3.3% [10], which was also in accordance with the VLBW incidence rate in Canada [11] and in India [12]. Furthermore, we noted that 57.4% of infants were considered to have a birthweight of 750-1000 g, and the rest were <750 g (42.6%). In a study from Spain, 37 children (21.8%) were classified as having ELBW (birth weight less than 1000 g), while 18.8% were classified as extremely preterm (gestational age less than 28 weeks) [13]. These findings indicate that even though the prevalence of ELBW is not high in many countries, it still exists. Improvements in perinatal care in recent years have significantly reduced the mortality and morbidity of ELBW infants.

Data from this study suggests that birthweight <750 g was more likely to be a product of multiple gestation and had a direct association with either twin or triplet fetuses. However, no significant link was observed between mortality and birth weight groups (750-1000 g vs. <750 g) (p=0.057). Contradicting this, Tchamo et al. found an increased risk for death, growth retardation, and delayed neurodevelopmental among surviving VLBW and ELBW infants [14]. In a study published in 2014, weight, height, and head circumference at the time of discharge were positively correlated with VLBW infants (p<0.001) [10].

Moreover, the lowest gestational age (23-26 weeks) and PPROM (>18 hours) had direct associations with birthweight 750-1000 g (p<0.001) but found no significant association between birth weight and other maternal factors, such as maternal age, number of parity, mode of delivery, maternal hypertension/preeclampsia, and maternal diabetes (p>0.05). This is not consistent with the study of Berger et al. [15]. Based on their accounts, a higher risk for preterm birth with early gestational age was positively associated with pre-pregnancy hypertension and pre-eclampsia. In contrast, preterm birth with late gestational age was correlated positively with maternal diabetes. This corroborated a study done in Germany by Spiegler et al., who reported that, based on regression estimates, hypertension and advanced maternal age increased the risk for preterm birth [16]. Also, they found that maternal hypertension was predicted to suffer a clinically specified preterm delivery, which may increase the chance of more VLBW infants with a modest growth restriction and fewer intraventricular hemorrhage in grades 3 or 4. Notwithstanding these reports, Anil et al. documented that maternal weight gain, preterm birth, and comorbidity during pregnancy were the significant risk factors for low birth weight [17]. 

Incidentally, we noted that increasing birthweight was positively correlated with maternal age, but increasing gestational age was inversely correlated with birthweight. This further suggests that every increase in maternal age will also likely increase infant birth weight; however, every increase in gestational age is directly correlated with a decrease in birth weight. Corroborating these reports, according to the adjusted regression model documented in Canada [18], late maternal age was found to be directly associated with preterm birth, and maternal age between 30 and 34 years was significantly associated with a lower risk of prematurity. In Nepal, the risk of low birth weight was more likely exhibited by mothers who had four or more antenatal (ANC) visits [19]. However, this was opposed by a study done in Burkina Faso by Lingani et al., wherein the frequency of ANC visits carried out by the mother during pregnancy was not identified as a protective factor for low birth weight [20].

This study has some limitations. First, the results of this study are not generalizable to the general population because it is a single-hospital-based study, and the data reflect the experience of a single institution. Therefore, extrapolations and interpretations of our results must be made with caution. Second, this study was retrospective in nature, with data for both mothers and infants extracted from charts and medical records. As a result, the study depended on the accuracy of existing documentation, and some maternal and infant information was unavailable for certain participants.

## Conclusions

Over the four-year period, the prevalence of ELBW was found to be relatively low in this study. Reduced gestational age and PPROM emerged as key risk factors for infants with birth weights of 750-1000 g, while multiple pregnancies, such as twins and triplets, were notably linked to birth weights below 750 g. Further investigation is warranted to better understand the prevalence of ELBW and to identify its contributing risk factors.
